# *In Vivo* Anti-HIV Activity of the Heparin-Activated Serine Protease Inhibitor Antithrombin III Encapsulated in Lymph-Targeting Immunoliposomes

**DOI:** 10.1371/journal.pone.0048234

**Published:** 2012-11-02

**Authors:** Mohammed Asmal, James B. Whitney, Corinne Luedemann, Angela Carville, Robert Steen, Norman L. Letvin, Ralf Geiben-Lynn

**Affiliations:** 1 Division of Viral Pathogenesis, Beth Israel Deaconess Medical Center, Boston, Massachusetts, United States of America; 2 Harvard Medical School, Boston, Massachusetts, United States of America; 3 New England Primate Research Center, Southborough, Massachusetts, United States of America; 4 Department of Genetics, Biopolymers Facility, Boston, Massachusetts, United States of America; 5 Acceleration BioPharmaceuticals, Inc., Woburn, Massachusetts, United States of America; University of South Carolina School of Medicine, United States of America

## Abstract

Endogenous serine protease inhibitors (serpins) are anti-inflammatory mediators with multiple biologic functions. Several serpins have been reported to modulate HIV pathogenesis, or exhibit potent anti-HIV activity *in vitro*, but the efficacy of serpins as therapeutic agents for HIV *in vivo* has not yet been demonstrated. In the present study, we show that heparin-activated antithrombin III (hep-ATIII), a member of the serpin family, significantly inhibits lentiviral replication in a non-human primate model. We further demonstrate greater than one log_10_ reduction in plasma viremia in the nonhuman primate system by loading of hep-ATIII into anti-HLA-DR immunoliposomes, which target tissue reservoirs of viral replication. We also demonstrate the utility of hep-ATIIII as a potential salvage agent for HIV strains resistant to standard anti-retroviral treatment. Finally, we applied gene-expression arrays to analyze hep-ATIII-induced host cell interactomes and found that downstream of hep-ATIII, two independent gene networks were modulated by host factors prostaglandin synthetase-2, ERK1/2 and NFκB. Ultimately, understanding how serpins, such as hep-ATIII, regulate host responses during HIV infection may reveal new avenues for therapeutic intervention.

## Introduction

Current HIV therapies employ combinations of small molecule inhibitors that target viral proteins at different steps in the HIV replication cycle in order to prevent the emergence of HIV resistance to therapy [1], [2], [3], [4]. Despite this strategy, resistance to one or more drug classes can emerge, resulting in a population of patients requiring salvage therapy [5]. The development of new anti-HIV therapeutics that target host proteins important for the virus life cycle could circumvent the problem of viral resistance. Host cell proteins that influence viral replication are less mutable than viral proteins, possibly offering an increased genetic barrier to the development of drug resistance. An analogous therapeutic concept has already proven efficacious in the treatment of HCV: stimulation of the host innate immune response using interferon-based therapy effectively blocks viral replication without induction of viral resistance [6].

Endogenous serine protease inhibitors (serpins) are part of the early innate immune response to viral infection that includes mannose binding lectins, soluble CD14, defensins and antimicrobial peptides [7]. The main biologic function of serpins is the blockage of protease activity involved in blood clotting and complement activation. Serpins belong to a superfamily of proteins that also regulate other inflammatory processes [8].

Serine protease inhibitors have a broad spectrum of anti-viral activity against HIV, HCV, HSV and the influenza virus [9], [10]. A number of clinical observations suggest a role for the serpins in controlling HIV infection and disease progression in the mucosa and the peripheral blood. For example, (1) there is a barrier to HIV transmission via the oral mucosa; this may be due to the anti-viral activity of Secretory Leukocyte Inhibitor (SLPI) in saliva [11]. (2) α_1_-anti-trypsin, the most abundant serpin in blood, prevents HIV replication *in vitro* at physiological concentrations; in addition, HIV replicates at a much higher rate in the blood of α_1_-anti-trypsin-deficient individuals, suggesting α_1_-anti-trypsin might reduce viral replication *in vivo* [12]. (3) The anti-HIV activity of α_1_-anti-trypsin is believed to be responsible for the relatively low transmission rates of HIV through contaminated needles, compared to that of HCV and HBV. (4) Furthermore, presence of the α_1_-anti-trypsin allelic variants M2 and A332A is associated with enhanced HIV-1 acquisition [13].

Antithrombin III (ATIII), a serpin with a role in the coagulation cascade, exhibits potent anti-HIV activity. ATIII exists in three different forms under physiological conditions. In its inactive latent (L) form, ATIII circulates with its reactive COOH-terminal loop not fully exposed, thereby preventing its binding to thrombin. Upon binding to heparin, ATIII undergoes a conformational change to an activated, or stressed (S) form (here also termed hep-ATIII) allowing the exposure of the reactive COOH-terminal loop thus increasing the binding of thrombin by 100-fold. The resultant ATIII-thrombin complex eventually dissociates with the release of thrombin and an ATIII with a cleaved reactive loop, inducing a conformational change of ATIII to a relaxed (R) form.

A proteolytically cleaved form of ATIII was originally discovered to be a CD8^+^ T cell anti-HIV factor (CAF) - a non-cytolytic innate immune response in HIV-1 long-term non-progressors [14], [15]. The S form of ATIII has greater antiviral activity against HIV and the simian immunodeficiency virus (SIV) than the R form; the L form has no anti-viral activity [14]. Hep-ATIII is up to 10-fold more potent at inhibiting HIV than the non-activated form of ATIII [16]. When compared to other serpins with anti-HIV activity, α1-antitrypsin and SLPI, heparin-activated antithrombin III (hep-ATIII) displays up to 10^6^ fold higher anti-HIV activity *in vitro* [11], [12], [14], [16], [17], [18]. The anti-viral activity of hep-ATIII and ATIII is mediated at least in part by host cell factors prostaglandin synthetase 2 (PTGS2) and transcription factor NFκB [9]. Two hundred-fold less hep-ATIII was required as compared to non-activated ATIII to elicit equivalent changes in gene transcription of these host cell factors [9].

In the present study, we sought to validate hep-ATIII as an HIV therapeutic using *in vitro*, humanized mouse and preclinical primate models of HIV infection. In order to evaluate the potential utility of ATIII as a salvage agent in patients with multidrug resistant HIV, we assessed the ability of hep-ATIII to inhibit a range of drug-resistant HIV-1 isolates *in vitro*, and in humanized mice infected with highly drug resistant HIV-1. In addition, we studied the effects of ATIII treatment in rhesus macaques chronically infected with SIV. In a novel therapeutic approach, we used anti-HLA-DR antibodies engrafted into immunoliposomes to encapsulate hep-ATIII (termed ET-ATIII): it has been shown that these immunoliposomes specifically target HLA-DR positive cells in lymph nodes including monocytes, macrophages and activated CD4^+^ T lymphocytes, allowing concentration of therapeutic ATIII in the main cellular reservoirs of HIV and SIV [19]. Finally, we sought to understand the mechanism by which hep-ATIII exerts its antiviral activity. We studied the gene expression profiles of peripheral blood mononuclear cells (PBMC) from SIV-infected macaques treated with hep-ATIII, and identified the transcriptional networks activated or repressed by hep-ATIII treatment. By elaborating the biologic networks associated with HIV inhibition by the innate immune system, we hoped to identify potential biomarkers of drug efficacy, as well as potential future drug targets.

## Materials and Methods

### Ethics Statement

All animal experimental protocols were approved by the Harvard Institutional Animal Care and Use Committee (IACUC) [20]. Human blood sampling was reviewed and approved by the Human Research Ethics Committee of the Beth Israel Deaconess Medical Center (BIDMC) and Harvard Medical School (IRB 2006-P-000004). Written consent for human blood collection was waived since no personal data were collected. Harvard Medical School follows NIH guidelines for animal handling and has Animal Welfare Assurance A3153-01 on file with the Office for Protection of Research Risks. The institutions involved in the studies maintain full accreditation from Association for Assessment and Accreditation of Laboratory Animal Care.

Adult rhesus macaques (*Macaca mulatta*) were housed at the New England Primate Research Center and Harvard Medical School, a primate animal facility that is accredited by the Association for the Assessment and Accreditation of Laboratory Animal Care International. Research was conducted in compliance with the Animal Welfare Act and other US federal statutes and regulations relating to animals and experiments involving animals, and adhered to principles stated in the Guide for the Care and Use of Laboratory Animals, National Research Council, 1996. All steps were taken to ameliorate the welfare and to avoid the suffering of the animals in accordance with the “Weatherall report for the use of non-human primates” recommendations. Animals were housed either socially or in adjoining individual primate cages allowing social interactions, under controlled conditions of humidity, temperature and light (12-hour light/12-hour dark cycles). Food and water were available *ad libitum*. Animals were fed commercial monkey chow and treats by trained personnel. Environmental enrichment consisted of commercial toys. Blood draws were conducted under sedation by trained personnel under the supervision of veterinarians.

### Mice

NOD/*scid/scid*-beta-2 microglobulin (b2m) knockout mice (Nod/Scid/b2m^null^ mice) (6–8 weeks) and C57BL/6 mice were from the Jackson Laboratory (Bar Harbor, ME).

### Source and Activation of ATIII

Recombinant human ATIII was produced in transgenic goats by GTC Biotherapeutics (Framingham, MA). These transgenic animals express human ATIII in their mammary glands and secrete it into their milk. ATIII was purified from goat milk through clarification through a 500-kDa tangential flow membrane filtration unit, captured and then eluted through a heparin affinity chromatography column. It was further purified by anion-exchange chromatography and hydrophobic interaction chromatography as described earlier [21]. The product had a biological activity of 6 U/mg. It was more than 99% pure, as determined by sodium dodecyl sulfate-polyacrylamide gel electrophoresis (SDS-PAGE) and by silver-staining, or by C4 high-pressure liquid chromatography (HPLC).

Anti-viral ATIII activity was activated by incubation with heparin as previously described and subsequently referred to as hep-ATIII. Briefly, ATIII was incubated with equal amounts (w/w) of heparin sodium (Polysciences, Warrington, PA, cat. no. 01491, 40–3 kDa fraction) overnight at 37°C to form a non-covalent ATIII-heparin complex. Unbound heparin was then removed by Sephacel 100 ÄKTA FPLC (GE Health Care Life Sciences, Piscataway, NJ) at 1 ml/min. Protein preparations resulted in less than 5% (w/w) free heparin measured by FPLC with refractive index detection and a formula described earlier [16]. Protein purity was determined by sodium dodecyl sulfate-polyacrylamide gel electrophoresis (SDS-PAGE) and silver staining using a Bio-Rad kit (Bio-Rad Life Science, Hercules, CA) with a 15% slab gel. Molecular weight was compared to a low-range protein molecular weight marker (Bio-Rad).

### Encapsulation of Hep-ATIII

To produce 10 ml sterically stabilized anti-HLA-DR unilamellar immunoliposomes a two step protocol was used: The first step included the derivatization of the anti-HLA-DR antibody: 0.6 ml of 0.1 M of octyl glucoside (OG) was added to 6 ml of MES/NaCl buffer. Then 3.30 mg of N-glutaryl-dioleoylphosphatidylethanolamine (NGPE) lipid (Sigma-Aldrich) was dissolved in 2 ml of chloroform and added to a 50 ml round bottom flask. Chloroform was evaporated in a rotary evaporator. OG in MES/NaCl buffer was added to the dried film. Then 1.1 ml of 0.25 M 1-ethyl-3-(3′-(dimethylamino)propyl)carbodiimide (EDC) (Sigma-Aldrich) and 1.1 ml of 0.1 M N-hydroxysulfosuccinimide (NHS) was be added and incubated at room temperature for 10 min, then adjusted to pH 7.5. Anti-HLA-DR antibody (50 mg, clone 2.06, IgG1, American Type Culture Collection (ATCC) dialyzed against sodium borate buffer was added. This solution was incubated for 12 h at 4°C and afterwards dialyzed against PBS buffer. The NGPE conjugated antibody was concentrated in a vacuum concentrator.

The second step included the encapsulation of hep-ATIII and binding of the antibody to the liposome: 40 mg of dioleoylphosphatidic acid (DOPA) (Sigma-Aldrich) and 164 mg of dioleoylphosphatidylethanolamine (DOPE) (Sigma-Aldrich) was dissolved in 100 ml of chloroform in a round bottom flask connected to a rotary evaporator. The chloroform was evaporated by vacuum until a thin lipid film was formed. The NGPE conjugated antibody and 1 ml hep-ATIII solution (20 mg/ml) was added to the dried lipid film. This film was hydrated overnight at 4°C during which liposomes formed. The liposomes were sized to 100 nm by 20 times extrusion through a 5-micron diameter pore polycarbonate membrane filter using a Lipex extruder (Northern Lipids, Burnaby, BC, Canada). The liposomes were dialyzed against 10 L of PBS buffer for 36 h. Efficiency of protein encapsulation was determined by measurement of retained protein in the supernatant after centrifugation of immunoliposomes by a bicinchoninic acid assay (BCA) (Pierce, Rockford, Ill). The phospholipids concentration was measured using visible derivative spectroscopy. The total amount of the lipids was 20 mg/ml. The total amount of encapsulated protein was 0.05 mg/ml and the total amount of the conjugated anti-HLA-DR antibody was 2.4×10^4^ mol/ml.

Conventional multilamellar liposomes were prepared as follows: Phosphatidylcholine (PC) and cholesterol (both purchased from Avanti Polar Lipids, Alabaster, AL) were dissolved in chloroform (total concentration of lipids in chloroform was around 2 mg/ml) and added to a 250 ml capacity round bottom flask. The chloroform was evaporated from the flask using a rotary evaporator for at least 4 h. A thin lipid film was formed on the wall of the round bottom flask. Hep-ATIII protein was purged with Argon gas for 10 min and added to the thin lipid film. The lipid film was hydrated for 4 h at 4°C. The liposomes were formed after the hydration. Liposomes were extruded 20 times through a 5 µm polycarbonate membrane filter using a Lipex extruder. The liposomes were centrifuged. The phospholipid concentration was measured spectophotometrically using visible derivative spectroscopy. The cholesterol was measured using the cholesterol measuring kit from Sigma-Aldrich. The amount of the encapsulated protein was measured by BCA as described above. The concentration of PC was 20 mg/ml and that of cholesterol was 5 mg/ml. The liposomes were 1–2 µm in size and encapsulated 0.8 mg/ml hep-ATIII.

### HIV-1 Env Pseudovirus Production and Titration

We used Env plasmids for HIV-1 pseudovirus production representing the standard panel of clade B clones (PVO.4, QH0692) and clade C clones (Du123.6, Du 151.2) from NIH AIDS Research and Reference Reagent Program (ARRRP). Stocks of single-round infectious HIV-1 Env pseudovirus were produced by cotransfecting 293T/17 cells (1.7×10^7^ cells per T75 flask) with 2 µg of an HIV-1 rev/env expression plasmid and 12 µg of an env-deficient HIV-1 backbone plasmid (pSG3ΔEnv) using Lipofectamine transfection reagent (Invitrogen, Grand Island, NY). Pseudovirus-containing supernatant was harvested 24 h following transfection and clarified by centrifugation and 0.45-µm filtration. Single-use aliquots (1.0 ml) were stored at −80°C. The 50% tissue culture infectious dose (TCID_50_) for each pseudovirus preparation was determined by infection of TZM.bl cells as previously described [22]. A T-cell-line-adapted (TCLA) strain of HIV-1 MN was obtained from the NIH ARRRP as contributed by R. Gallo [23], [24], and cell-free stocks were generated using H9 cells as previously described [25].

### HIV-1 Pseudovirus Inhibition Assay

Virus inhibition was measured using a luciferase-based assay in TZM.bl cells as previously described [26]. This assay measures the reduction in luciferase reporter gene expression in TZM.bl cells following a single round of virus infection. Briefly, TZM.bl cells were added (1×10^4^/well in a 100-µl volume) in 10% D-MEM growth medium containing DEAE-dextran (Sigma-Aldrich) at a final concentration of 11 µg/ml. Three-fold serial dilutions of 250 µg/ml non-activated ATIII, 50 µg/ml heparin, 15 µg/ml hep-ATIII and 15 µg/ml 135 kDa ATIII complex stock solution were performed in triplicate (96-well flat bottom plate) in 10% D-MEM growth medium (100 µl/well) and added to the cells. An amount of 200 TCID_50_ of virus was then immediately added to each well in a volume of 50 µl, and the plates were incubated for 48 h at 37°C. Assay controls included replicate wells of TZM.bl cells alone (cell control) and TZM.bl cells with virus (virus control). Following incubation period, 150 µl of assay medium was removed from each well and 100 µl of Bright-Glo luciferase reagent (Promega, Madison, WI) was added. The cells were allowed to lyse for 2 min, and then 150 µl of the cell lysate was transferred to a 96-well black solid plate, and luminescence was measured using a Victor 3 luminometer (Perkin Elmer, Hopkinton, MA). The 50% inhibitory concentration (IC50) titer was calculated as the serum dilution that caused a 50% reduction in relative luminescence units (RLU) compared to the level in the virus control wells after subtraction of cell control RLU.

### *In vitro* Acute HIV Infection Assay Using Primary Isolates

For *in vitro* assays, human peripheral blood mononuclear cells (hPBMC) from HIV-1-seronegative donors were obtained by Ficoll-Hypaque gradient centrifugation of heparinized whole blood. After 3 days of mitogen stimulation (6.25 µg/mL concanavalin A), hPBMC were re-suspended at a concentration of 1×10^5^ cells/ml in RPMI 1640 culture medium (Sigma-Aldrich) supplemented with 10% fetal calf serum (Sigma-Aldrich), penicillin (50 U/ml), streptomycin (50 µg/ml), L-glutamine (2 mM), HEPES buffer (10 mM), and 50 U/ml interleukin-2 in 24-well tissue culture plates (Becton Dickinson, San Jose, Ca). An HIV-1 inoculum of 1,000 50% tissue culture infective doses (TCID)/10^5^ cells was added to the hPBMC for 2 h at 37°C and cells were washed extensively. Hep-ATIII, conventional liposomes and sterically-stabilized anti-HLA-DR immunoliposomes encapsulating hep-ATIII were added in serial dilutions at day 1 and day 4. Fifty percent of medium was replaced at day 4. Each condition was tested in triplicate. To determine viral inhibition, cell-free culture supernatants were harvested and analyzed by an enzyme-linked immunosorbent assay (ZeptoMetrix Corporation, Buffalo, NY) for HIV-1 p24 antigen on day 7 of culture and compared against a vehicle control. Different drug concentrations were used in a virus-specific cell-based assay to measure inhibition. From these data, the IC50, was calculated using the MacSynergy II Software [27]. Controls for inhibition experiments included vehicle buffer, bovine serum albumin (up to 30 µM) and a heparin only control. Additionally, for the liposome inhibition assays, empty liposomes were used as controls. Controls never reached more than 25% inhibition compared to untreated controls. The new integrase inhibitor 118-D-24, belonging to the azido-containing diketo acid derivates, was used as a control of an anti-HIV drug with a known IC50 between 2 and 10 µM [28].

### Treatment of Rhesus Macaques with Different Forms of ATIII

For the non-human primate studies, Indian-origin rhesus macaques were intravenously infected with a 50-fold 50% monkey infectious dose (MID_50_) of SIV_mac251_, and followed for more than 450 days after infection. Animals then received 0.8 µmol/kg, non-activated ATIII by the intravenous route daily for 4 days and then every 3 days for another 9 days. Hep-ATIII was administered daily for the first 4 days at 0.6 µmol/kg. Immunoliposome preparations were injected as 1.5 ml subcutaneous administrations at day 1 and 2 with 0.3 nmol/kg hep-ATIII.

### Measurements of SIV Load

To measure SIV load, viral RNA was isolated from 200 µL of plasma using the NucliSENS Isolation Kit (Biomerieux, Lyon, France) according to the manufacturer’s protocol. RNA was reverse transcribed in parallel with an SIV-*gag* RNA standard using the gene-specific primer sGag-R 5′CACTAGGTGTCTCTGCACTATCTGTTTTG-3′ with the following conditions: the 50 µL reactions containing 1×buffer (250 mM Tris-HCL pH 8.3, 375 mM KCl, 15 mM MgCl2), 0.25 µM primer, 0.5 mM dNTPs (Roche), 5 mM dTT, 500 U Superscript III RT (Invitrogen, Carlsbad, CA), 100 U RNaseOUT (Invitrogen), and 10 µL of sample. RT conditions were 1 h at 50°C, 1 h at 55°C and 15 minutes at 70°C. Samples were treated with RNase H (Stratagene, La Jolla, CA) for 20 min at 37°C. For the real-time PCR (RT-PCR) reactions the EZ RT-PCR Core Reagents (Applied Biosystems, Foster City, CA) were used according manufacturer’s protocol. Primer sequences were used as described previously [29] with forward primer s-Gag-F, 5′-GTCTGCGTCATCTGGTGCATTC-3′, reverse primer s-Gag-R, 5′-CACTAGGTGTCTCTGACTATCTGTTTTG-3′, and the probe s-Gag-P, 5′-CTTCCTCAGTGTGTTTCACTTTCTCTTCTGCG-3′, linked to Fam and BHQ (Invitrogen). All reactions will be carried out on a 7300 ABI RT-PCR system (Applied Biosystems) in triplicate according to the manufacturer’s protocols.

### Murine Model of HIV-1 Infection

Protection from HIV induced cytotoxicity in hPBMC was measured in an acute HIV-1 infection model with 6 to 8-week old female Nod/Scid/b2m^null^ mice from The Jackson Laboratory (Bar Harbor, ME). These mice were chosen because of their lack of murine lymphoid cells in the spleen and superior engraftment of hPBMC compared to other Nod/Scid mice [30]. This allows for the quantification of HIV-induced cytotoxicity in engrafted hPBMC in mice as splenocytes are largely of human origin. For Nod/Scid/b2m^null^ mice grafts, 10^7^ freshly isolated hPBMC were acutely infected *in vitro* with a multi-drug resistant HIV-1 clone (GenBank no. AY351719, NIH no.7324-4) [31] at 1000 TCID_50_/ml and incubated at 37°C for 2 h. This primary HIV-1 isolate showed 3.5-fold resistance to abacavir (ABC), 1.7-fold resistance to didanosine (DDI), 3.6-fold resistance to lamivudine (3TC), 2.3-fold resistance to stavudine (D4T), 5.2-fold resistance to tenofovir (TDF), 1.4-fold resistance to zalcitabine (DDC), and 464-fold resistance to zidovudine (AZT) as determined by the Virologic PhenoSense™ Assay [31]. Cells were washed of excess virus and 3×10^6^ hPBMC were administered by the intraperitoneal route into the Nod/Scid/b2m^null^ mice. Twenty-five nmol/kg of hep-ATIII was administered by intravenous route via the tail vein once daily. Mice spleens were harvested after 14 days of hep-ATIII treatment, splenocytes were isolated and red blood cells were lysed with BD Pharm Lyse™ lysing solution (BD Bioscience, Bedford, MA). Splenocytes were counted under a light microscope after Trypan Blue exclusion staining.

### Measurement of *in vitro* Cytotoxicity

To test for cytotoxicity in the *in vitro* inhibition assays, uninfected drug-treated cytotoxicity controls were maintained at the highest concentration of drug tested, and assessed by Trypan Blue dye exclusion and Neutral Red staining. Additionally, viability of cells was tested at day 5 after drug addition with the Guava Technologies EasyCyte Plus Flow Cytometer system (Guava Technologies, Hayword, CA). The Guava ViaCount® Assay was used to test for apoptotic cells.

### Measurement of *in vivo* Cytotoxicity

Mice (C57BL/6, 5 per group) were injected with different amounts of hep-ATIII loaded immunoliposomes. Weight, CBC and blood chemistries were measured.

### Affymetrix GeneChip® Rhesus Macaque Genome Array, Gene-expression and Network Analysis

To measure the effect of hep-ATIII on gene expression, rhesus macaque PBMC were purified from the blood of hep-ATIII treated monkeys and vehicle treated controls. Total RNA from cells was purified after shredding cells using the QIAshredder homogenizer (Qiagen, Valencia, CA) with RNAeasy spin-columns (Qiagen) according to manufacturer protocol. Integrity and concentration of RNA samples was tested using an Agilent BioAnalyser and applied to an Affymetrix system setup consisting of target preparation, target hybridization, probe array washing, staining and probe array scan. The Affymetrix GeneChip® Rhesus Macaque Genome Array was used to analyze gene-expression patterns activated by hep-ATIII treatment. This array enables whole genome gene-expression measurement of 47,000 rhesus macaque transcripts. Gene expression and protein network analysis was performed using the Ingenuity 8.0 software (Ingenuity Systems, Redwood City, CA).

### Complete Blood Count and Peripheral Blood CD4_+_ T Lymphocyte Count for Rhesus Macaques and C57BL/6 Mice

Complete Blood Count (CBC) testing white blood cell count (WBC), red blood cells (RBC), hemoglobolin (HGB), hematocrit (HCT), mean red cell volume (MCV), mean cell hemoglobin (MCH), platelet count (PLT) and mean platelet volume (MPV) was performed using the ADVIA Hematology System (Bayer, Leverkusen, Germany) according manufacturers protocol. Peripheral blood CD4^+^ T lymphocyte counts were calculated by multiplying the total lymphocyte count by the percentage of CD3^+^CD4^+^ T cells determined by mAb staining and flow cytometric analysis [32].

### Data Analysis

The statistical significance of differences between groups was determined using the program GraphPad Prism 4.0 (GraphPad Software, La Jolla, CA). A *P* value of <0.05 was considered statistically significant. Statistical analysis was performed by use of the Mann-Whitney test, paired T-test and the ΔΔC_t_ method. Error bars represent standard error of the mean (S. E.).

## Results

### Anti-viral Activation of ATIII

**Figure 1:**
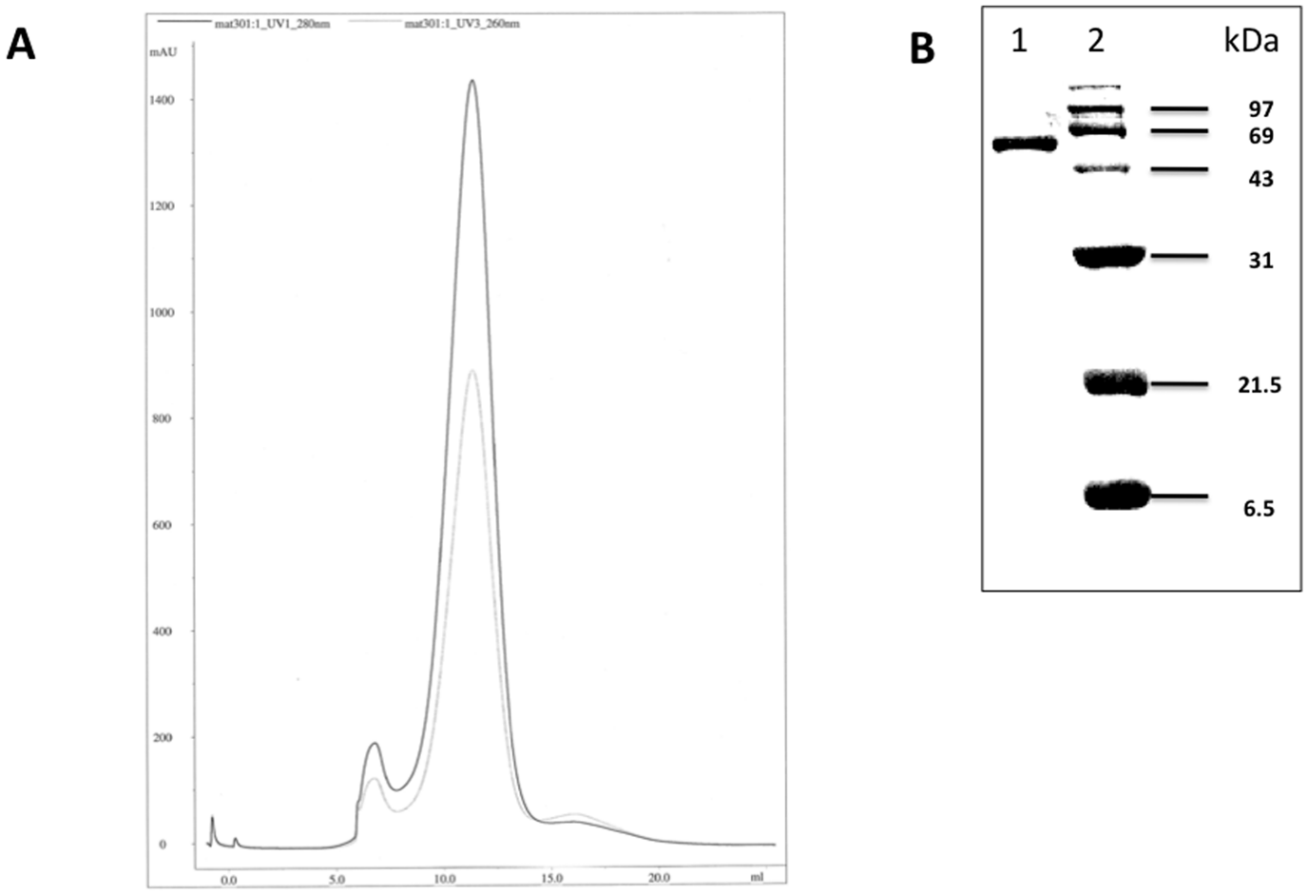
Characterization of heparin-activated ATIII. (**A**) Sepharcyl S100 ÄKTA FPLC of purified hep-ATIII. A 9–14 ml fraction was separated, termed as hep-ATIII and used for our experiments. Detection at 260 nm for protein detection and 280 nm for heparin detection is shown. (**B**) Hep-ATIII purity and molecular weight were also determined by SDS-PAGE and silver staining (Bio-Rad kit) of a 15% slab gel. For molecular weight determination a low-range protein molecular weight marker (Bio-Rad) was used. Lane 1∶2 µg hep-ATIII; lane 2: phosphorylase B (97 kDa), serum albumin (69 kDa), carbonic anhydrase (31 kDa), trypsin inhibitor (21.5 kDa), aprotinin (6.5 kDa).

Anti-viral capacity of ATIII was activated by overnight incubation with heparin at 37°C. This resulted in a mixture containing three compounds, which we subsequently separated by Sephacyl S100 FPLC. We found 1–3% w/w of a 135 kDa ATIII polymer with a retention volume of 7–8 ml. Ninety-five to ninety-nine % (w/w) of the mixture was 66–68 kDa hep-ATIII, in which heparin was bound to ATIII, at a retention volume of 10–14 ml. At approximately 35 kDa a protein-free fraction comprised of unbound heparin polymer was contained (15–19 ml retention volume) **(** **Fig. 1A** **)**. The amount of this free heparin was calculated to be routinely below 5% (w/w) of the total mixture. We used fractionated hep-ATIII for our experiments.

We confirmed the purity of our fractionated hep-ATIII (66–68 kDa) preparations by SDS-PAGE and silver staining. We found that these hep-ATIII fractions were routinely more than 99% pure **(** **Fig. 1B** **)**.

### Anti-viral Activity of Activated and Non-activated ATIII in a Single-round HIV Pseudovirus Inhibition Assay

**Figure 2:**
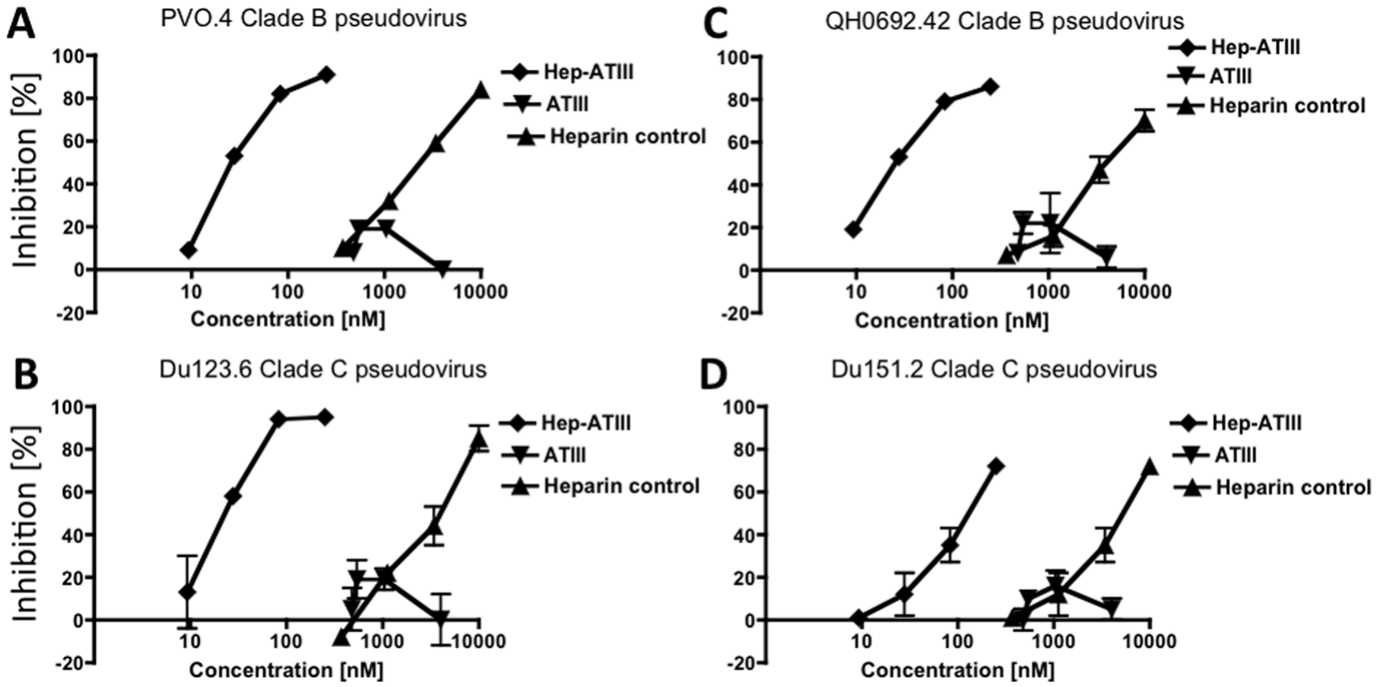
Effect of heparin-activated ATIII in pseudovirus inhibition assay. Pseudoviruses with clade B envelopes (in **A** and **C**) and clade C envelopes (in **B** and **D**) were treated with three fold dilutions of heparin-activated ATIII (hep-ATIII), unmodified ATIII and heparin. Percentage of inhibition was calculated by comparing of residual luciferase activity and untreated control. Experiments were done in triplicates. Data are shown as mean ± S.E.

We used the single-round HIV inhibition assay to measure the anti-viral activity of activated ATIII, hep-ATIII, and compared that with unmodified ATIII, the 135 kDa ATIII complex and a heparin control. This system allowed the measurement of inhibition effects on all phases in a virus’ life-cycle during one round of replication. To investigate the susceptibility of different HIV-1 envelopes to hep-ATIII inhibition we used pseudoviruses with clade B (PVO.4, QH0692.42) and clade C (Du123.6, Du151.2) envelopes. We found that the 66–68 kDa fraction, containing heparin-activated ATIII, exhibited anti-viral activity. The IC50 of hep-ATIII was between 20–100 nM, and independent of envelope usage **(** **Fig. 2** **)**. Unmodified ATIII had no inhibitory activity **(** **Fig. 2** **)** as well as the 135 kDa ATIII polymer (data not shown). Both demonstrated no inhibition as defined as inhibition below 25% in the pseudovirus inhibition assay. We also observed that heparin had an IC50 of 8 µM **(** **Fig. 2** **)**, comparable to what has been previously reported.

### Anti-viral Activity of Activated and Non-activated ATIII in PBMC Acutely Infected with Drug-resistant HIV-1

Anti-HIV-1 therapy that targets viral proteins must contend with the virus’ ability to rapidly evolve leading to the emergence of drug resistant isolates. Antiviral therapy that targets host proteins while stimulating host innate immunity may be able to circumvent antiviral resistance. We have previously shown that non-activated ATIII exerts limited anti-HIV-1 activity and has only limited effects on host cell gene expression. The anti-HIV-1 activity of ATIII can be augmented through overnight incubation with heparin [16], which results in non-covalent attachment of heparin to ATIII, creating activated hep-ATIII. We sought to determine if hep-ATIII could overcome current limitations in drug treatment options for multidrug resistant HIV.

We tested the anti-HIV activity of hep-ATIII against an array of primary isolates from different clades, with T-cell tropism (X4), macrophage tropism (R5) or dual-tropism (X4R5), and differing in drug resistance profiles. After infection of hPBMC with the various HIV isolates, we then added hep-ATIII at the indicated concentrations (at day 1 and 4 of infection), and followed the infection by p24-antigen ELISA for 7 days. We then used the resulting dose-response curves for day 7 to calculate IC50 values. Our experiments demonstrated that hep-ATIII exhibited potent anti-HIV activity that was independent of prior drug exposure, clade or co-receptor usage (**Table 1**). Importantly, we found that the anti-viral activity of hep-ATIII was observed at therapeutically favorable levels with IC50 values ranging from 16–240 nM (**Table 1**), which was similar to those observed in the pseudovirus inhibition assays **(** **Fig. 2** **)**.

**Table 1:** IC50 of hep-ATIII for drug resistant HIV-1

HIV-1 isolate	Co-receptor	IC50 (nM)
92/TH/006	R5	16
M46I/L63P/V82T/I84V^a^	R5X4	45
G762-3^b^	R5X4	53
RF	R5X4	74
RF/V82F/I84V^c^	R5X4	107
G691-6^d^	R5X4	205
N119	X4	213
10076-4^f^	R5X4	238

a resistant to the structurally diverse protease inhibitors: MK-639, XM323, A-80987, Ro 31-8959, VX-478, SC-52151 [70].

b AZT resistant, pre-drug [71].

c protease resistant derived from HIV-1 RF [72].

d AZT resistant, post-drug [71].

e Nevirapine resistant [73].

f multi-drug resistant HIV-1, fold resistance as determined by the ViroLogic PhenoSense™ Assay [15], [31]: ABC: 3.8; DDI: 1.6; 3TC: greater than upper limit of assay detection; D4T:1.9; TDF:1.3; DDC:1.8, AZT:6.3.

### Effect of Hep-ATIII on HIV-induced Cytotoxicity in Engrafted hPBMC in Nod/Scid/b2m^null^ Mice

**Figure 3:**
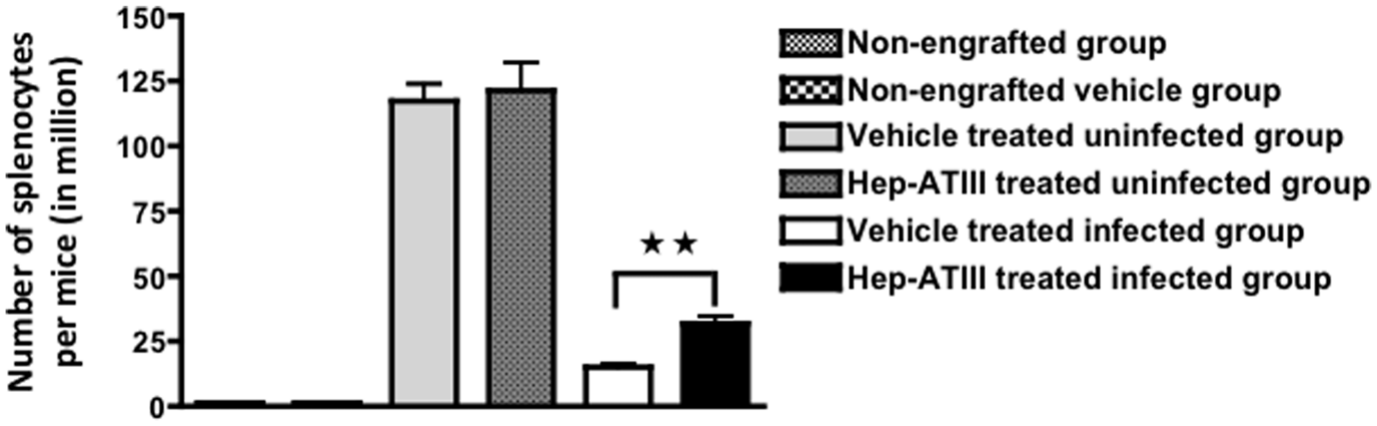
Effect of heparin-activated ATIII on HIV-induced cytotoxicity in NOD/Scid/b2m^null^ mice. (**A**) To measure HIV-induced cytotoxicity, splenocytes from NOD/Scid/b2m^null^ mice engrafted with HIV-infected hPBMC were quantified after 14-day incubation. hPBMC were purified by Ficoll-Hypaque gradient centrifugation, acutely infected at an MOI of 0.001 with a multidrug resistant HIV clone (GenBank no. AY351719, NIH no.7324-4) and incubated *in vitro* at 37°C for 1 hour. 3.5×10^6^ acutely infected hPBMC were administered via the peritoneal route into NOD/Scid/b2m^null^ mice (n = 5). These mice were then treated with a daily intravenous dose of 25 nmol/kg hep-ATIII. Splenocytes were counted under a light microscope after Pharma Lyse™ treatment and Trypan Blue exclusion staining. Controls included non-engrafted animals (with or without vehicle buffer treatment), uninfected animals not treated with hep-ATIII (vehicle uninfected control) and animals treated with hep-ATIII (hep-ATIII uninfected control). **, *P*<0.01, Mann-Whitney test. Data are shown as mean ± S.E.

We hypothesized that the immunomodulatory effects of hep-ATIII might not only inhibit HIV replication in target cells, but may also protect infected cells and uninfected bystander cells from HIV-related cytotoxic effects. We utilized the hPBMC engrafted, HIV-infected Nod/Scid/b2m^null^ mice to test this hypothesis. These mice lack murine lymphoid tissue and NK cell function, enabling superior engraftment of hPBMC compared to other Nod/Scid mice [30]. To simulate therapy of the challenging multidrug resistant HIV patient, we engrafted mice with hPBMC that were infected *in vitro* with a highly resistant HIV-1 isolate. We assessed whether daily treatment with low doses of hep-ATIII (25 nmol/kg) might reduce virus-induced hPBMC cytotoxicity. We used 5 Nod/Scid/b2m^null^ mice per non-engrafted group, non-engrafted vehicle group, vehicle treated uninfected group, hep-ATIII treated uninfected group, vehicle treated infected group and hep-ATIII treated infected group **(** **Fig. 3** **)**. We utilized a strain of HIV-1 that produced more than 90% virus-associated cell death of infected hPBMC in mice**.** We found that there was a 100% increase in splenocyte number after 14 days of treatment with hep-ATIII (*P* = 0.008, Mann-Whitney test) (**Fig. 3**) compared to untreated HIV-infected hPBMC engrafted control mice, suggesting protection of hPBMC from HIV cytotoxicity.

This demonstration of *in vivo* efficacy of hep-ATIII against HIV in the humanized mouse model prompted us to further test hep-ATIII in a more sophisticated non-human primate model of chronic lentiviral infection.

### Activity of Heparin-activated and Non-activated ATIII in Rhesus Macaques

Although there are numerous reports describing the *in vitro* anti-viral activity of the serpins α1-anti-trypsin [12], [33] and SLPI [11], [17], [18], there are no reports demonstrating their *in vivo* anti-viral efficacy. One obstacle to the *in vivo* assessment of the therapeutic potential of serpins may be that of generating and delivering an optimally activated form of the protease inhibitors. We utilized the rhesus macaque model of simian immunodeficiency virus (SIV) infection to assess whether variably activated forms of the serpin ATIII could inhibit viral replication. We have previously demonstrated that SIV strains are inhibited to a similar degree by ATIII as HIV-1 isolates [14].

Indian-origin rhesus macaques (*Macaca mulatta*) were infected by the intravenous (i. v.) route with 50-fold MID_50_ of SIV_mac251_ and were followed clinically for more than 450 days post infection. Viral loads of animals at the time of drug administration were stable with not more than 0.25 log_10_ variation between measurements in any given animal, and with viral loads routinely between 10^4^–10^6^ copies per ml for each of the different animals. Peripheral blood CD4^+^ T lymphocyte counts were between 10^4^ and 10^5^ cells per ml.

**Figure 4:**
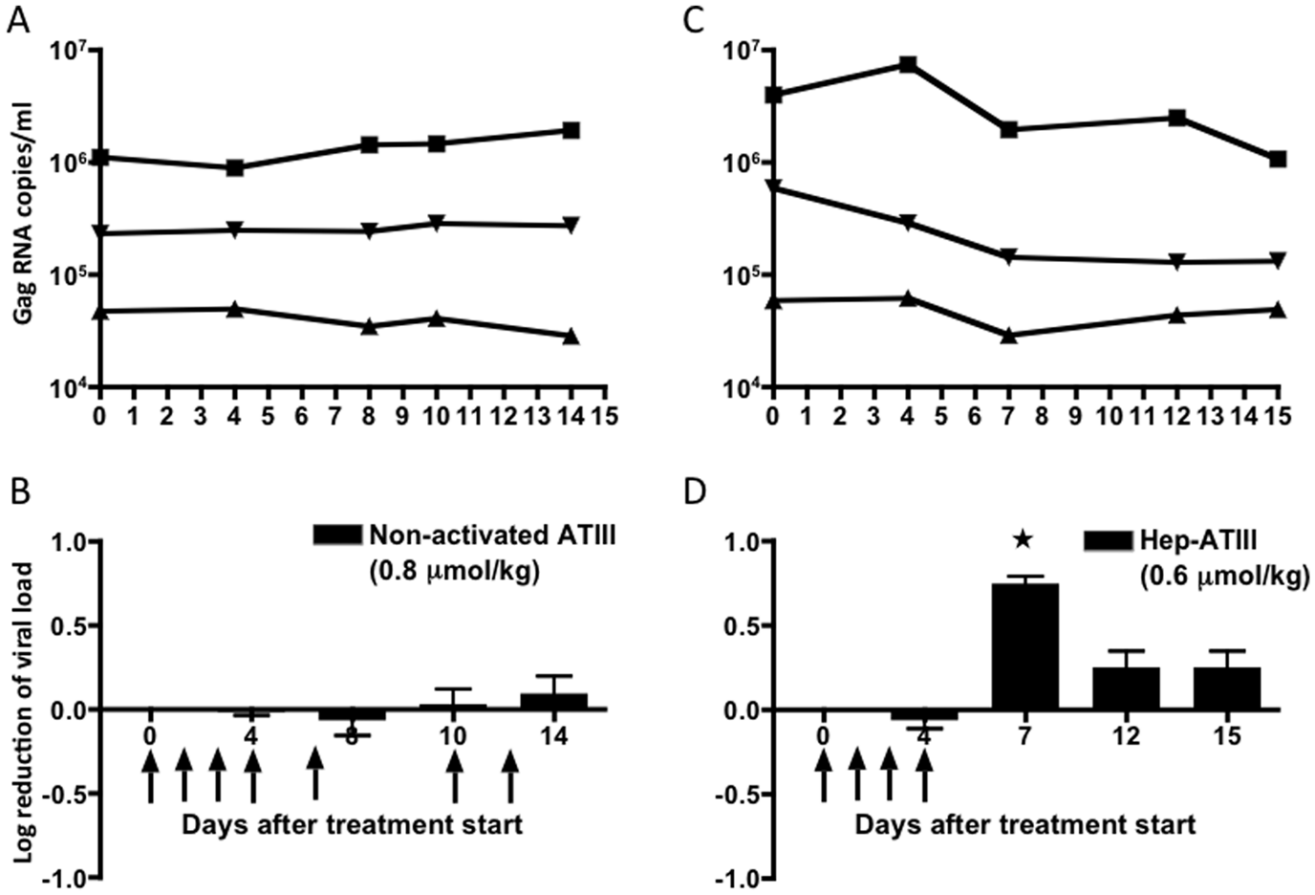
*In vivo* anti-viral activity of non-activated and heparin-activated ATIII in rhesus macaques. (**A**) Viral loads as RNA copies/ml of chronically SIV_mac239_ infected rhesus macaques treated with 0.8 µmol/kg non-activated ATIII (n = 3) and (**B**) corresponding log_10_ reduction of viral load of same treatment group. (**C**) Viral loads as RNA copies/ml of chronically SIV_mac239_ infected rhesus macaques treated with 0.6 µmol/kg heparin-activated ATIII (n = 3) and (**D**) corresponding log_10_ reduction of viral load of same treatment group. Administration via i. v. inoculation is shown at indicated time points depicted by arrows. Viral load was measured and compared to animals before treatment (day 0). *, *P*<0.05, paired T-test, compared to pre-treatment (day 0). Data are shown as mean ± S.E.

We first tested non-activated ATIII in 3 rhesus macaques. We based our dosing regimen for non-activated ATIII on a baboon model of sepsis, in which ATIII administration between 10 to 20-fold the physiological level (2.4 µM) was required to initiate an anti-inflammatory response to prevent disseminated intravascular coagulation [34]. A comparable dose had also been shown to be non-cytotoxic in primates [35]. Based on this, 0.8 µmol/kg ATIII was injected daily for 4 days during a loading phase and then applied every 3^rd^ day for a 14-day period. This administration schedule was chosen to prevent a significant decline in effective plasma drug concentration during the treatment period based on the 2.5–4.8 day half-life of ATIII measured by biologic and immunologic assays [36], [37], [38]. We confirmed lack of toxicity by both CBC and blood chemistries (data not shown). Although ATIII was administered at supra-physiologic doses we could not detect anti-viral activity as measured by changes in plasma viral RNA levels (**Fig. 4A** **/B**).

Hep-ATIII anti-viral activity is up to more than 10-fold more potent than that of ATIII [16]. As our animals appeared to tolerate the dose of non-activated ATIII without side effects, we administered a comparable dose of hep-ATIII for four days. Hep-ATIII exhibits a reduced half-life of 2 days [36], [37], [39]. From these data we estimated that daily dosing for 4 days with 0.6 µmol/kg will produce serum concentrations above 10 µM, more than 4-fold the physiologic concentration of ATIII. Using 3 rhesus macaques, we observed a 0.9 log_10_ reduction in plasma viral load seven days after the first administration (day 7) of hep-ATIII, amounting to an 80% reduction in circulating virus (*P* = 0.004, paired T-test). The viral RNA reduction persisted for 11 days after the discontinuation of the hep-ATIII treatment (**Fig. 4C** **/D**). Thus, we demonstrate an *in vivo* therapeutic effect, and that this serpin activity is dependent on proper biochemical activation.

### *In vitro* Anti-viral Activity of Hep-ATIII after Encapsulation into Sterically Stabilized Anti-HLA-DR Immunoliposomes

In addition to proper biochemical activation, another obstacle to the successful therapeutic use of serpins may be in their pleiotropic activity, and potential for negative off-target side effects. We hypothesized that by targeting hep-ATIII specifically to tissues in which HIV replication is most robust, we could simultaneously increase therapeutic efficacy and reduce off-target drug exposure. We thus sought to determine if the therapeutic potential of hep-ATIII may be enhanced by encapsulation of hep-ATIII into immunoliposomes. As serpins lack the ability to specifically traffic to the lymph nodes, the primary tissue of viral replication, we sought to facillitate delivery of hep-ATIII to the lymph nodes by encapsulation into sterically stabilized anti-HLA-DR immunoliposomes (termed ET-ATIII) [40]. These liposomes enable dissemination of drugs directly to the lymphatic system, and also primarily home in on HIV-1 infected cells expressing the HLA-DR receptor [40].

We assessed the viral inhibitory activity of unencapsulated hep-ATIII, hep-ATIII encapsulated into conventional liposomes lacking anti-HLA-DR, and ET-ATIII against the dual-tropic (X4R5) HIV 89.6, and the macrophage-tropic (R5) SF162 in cell culture. These two HIV-1 isolates are derived from two different viral reservoirs: 89.6 was isolated from PBMC, and SF162 from the cerebrospinal fluid. 89.6 is a prototype for syncytium-forming, highly cytopathic HIV-1 viruses that replicate to high titers in cells bearing either CXCR4 or CCR5 receptors, reflective of isolates that may be found in individuals with AIDS [41]. In contrast, SF162 has more restricted co-receptor usage, is less cytopathic and more typical of primary, transmitted HIV isolates [42].

We infected hPBMC with SF162 and 89.6, and treated cells with hep-ATIII either unencapsulated or encapsulated into either conventional liposomes or sterically-stabilized anti-HLA-DR immunoliposomes (ET-ATIII) (**Table 2**). We found that encapsulation into immunoliposomes increased the anti-viral potency of hep-ATIII 107–150–fold, resulting in sub-nanomolar IC50 values of 0.4–0.7 nM. In contrast the use of conventional liposomes had only a limited effect on hep-ATIII potency. The IC90 for immunoliposomes was 3.7 nM for the 89.6 strain and 4.1 nM for the SF162 strain. We also compared the anti-HIV activity of ET-ATIII to that of the integrase inhibitor, 118-D-24. We observed an IC50 for 118-D-24 of 1750 nM, similar to what has been previously reported [28], suggesting a 1000-fold greater activity for ET-ATIII on a molar basis (**Table 2**). A vehicle liposome control was used for both liposome constructs to confirm that the liposomal vehicle did not have antiviral properties itself, consistent with prior studies [43].

**Table 2:** IC50 of hep-ATIII in different liposome formulations.

Formulation	HIV-1 isolate	IC50 (nM)
Free hep-ATIII control	89.6	60
	SF162	75
Conventional Liposomes	89.6	24
	SF162	22
ET-ATIII	89.6	0.4
	SF162	0.7

Conventional liposomes and sterically-stabilized immunoliposomes have been used to encapsulate imaging reagents, small molecule drugs and proteins in both pre-clinical experiments and clinical settings, including in HIV patients, to minimize drug side-effects by increasing target tissue specificity [19], [40], [43], [44], [45], [46], [47]. There has been no cell toxicity reported after administration of proteins encapsulated in conventional or sterically stabilized liposomes [43], [48], [49]. To assess the therapeutic index (TI) of our liposomes we tested for cytotoxicity over a wide dose range. We calculated TI as the ratio of the 50% cytotoxic dose (CD50) to the IC50. To measure the CD50 we tested the effect of ET-ATIII on cell viability using the Guava ViaCount® Assay. For these assays we used hPBMC from an HIV-1 infected patient as the indicator of cytotoxicity, as these best reflect cell populations that would be targeted by the liposome treatment *in vivo*. Additionally, we also assessed the toxicity of the liposomes against endothelial cells as ATIII is known to affect this cell type, inducing the release of anti-inflammatory prostacyclin and prostaglandins [7], [8]. We found no significant decrease in viability of either cell population in response to an escalating dose of encapsulated hep-ATIII (3–30 nM). We found that encapsulated hep-ATIII had a very favorable TI of >100. A TI of >10 is considered feasible for an antiviral drug.

### *In vivo* Anti-viral Activity of hep-ATIII after Encapsulation into Sterically Stabilized Anti-HLA-DR Immunoliposomes

**Figure 5:**
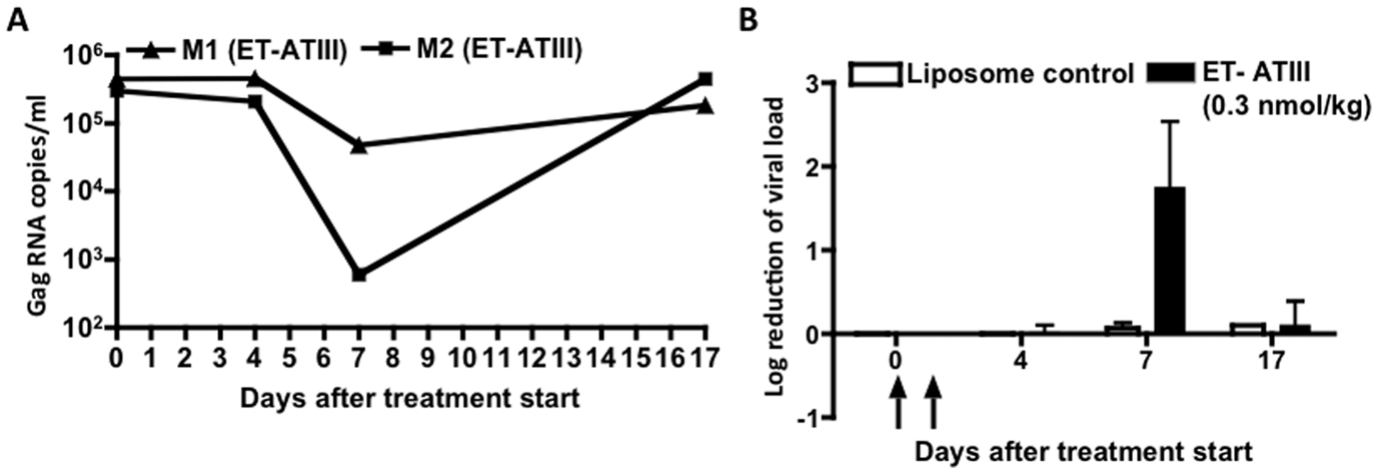
*In vivo* anti-viral activity of ET-ATIII in rhesus macaques. Chronically SIV_mac239_ infected rhesus macaques (n = 2) were treated with 1.5 ml ET-ATIII (0.3 nmol/kg encapsulated hep-ATIII) at indicated time points depicted by arrows. (**A**) Viral load of ET-ATIII treated animals as RNA copies/ml. (**B**) Log_10_ reduction of viral load. Vehicle liposomes were used as a control. Viral load was measured and compared to animals before treatment (day 0). Data are shown as mean ± S.E.

Under normal physiologic conditions, ATIII is not detectable in the lymphatic system [50]. As the lymph nodes are a major compartment for HIV-1 replication, we hypothesized that targeting hep-ATIII directly to the lymphatic system may increase the *in* *vivo* activity of hep-ATIII in our chronically SIV-infected macaque model. It has been previously shown that sterically stabilized anti-HLA-DR immunoliposomes accumulate in cervical and brachial lymph nodes, suggesting that they may effectively target HLA-DR positive cells, i.e. monocytes/macrophages and activated CD4^+^ T lymphocytes that are the primary cellular targets of HIV-1 [19]. Hep-ATIII was encapsulated in immunoliposomes (0.05 mg/mL) of 100 nm diameter with 4×10^4^ mol/ml anti-HLA-DR antibody incorporated. We subcutaneously injected chronically two SIV-infected animals with 0.3 nmol/kg of ET-ATIII. The overall quantity of hep-ATIII inoculated was 2000-fold less than the dose of unencapsulated hep-ATIII used in our previous *in vivo* experiments. We determined this dose based on two prior findings: firstly, our *in vitro* inhibition data suggested that ET-ATIII was at least 23-fold more effective than hep-ATIII compared to the lowest IC50 found for the resistant strains **(** **Table 1** **)**; secondly, prior reports have suggested a 100-fold increase of efficacy in anti-HIV-1 drugs activity when these are delivered directly to lymph tissue [40]. We observed that ET-ATIII decreased plasma SIV viral RNA by an average of 2 log_10_ (range 0.97–2.5 log_10_) at day 7 **(** **Fig. 5A** **/B)** - 5 days after the 2^nd^ treatment, demonstrating the potent anti-viral activity of ET-ATIII. Control immunoliposomes did not alter viral load (**Fig. 5B**).

To further investigate the limits of safe dosing of ET-ATIII, we used 5 wild type C57BL/6 mice per group to assess for systemic toxicity of high doses of ET-ATIII. We tested concentrations of ET-ATIII up to 30 nmol/kg, 100-fold the effective dose for *in vivo* SIV inhibition. We measured the complete blood count (CBC) to assess for the hematologic effects of the ET-ATIII formulation. In particular, the hematocrit (HCT) was used as a surrogate marker for internal bleeding, a potential side effect of the use of hep-ATIII. We found no significant change in animal weight, white blood cell count or HCT. All other blood parameters as well as liver function tests were within the expected normal range (data not shown). Thus, no *in vivo* cytotoxicity could be detected in mice at concentrations significantly in excess of the therapeutically effective anti-viral dose.

### Gene Expression in PBMC from Hep-ATIII Treated Rhesus Macaques

**Figure 6:**
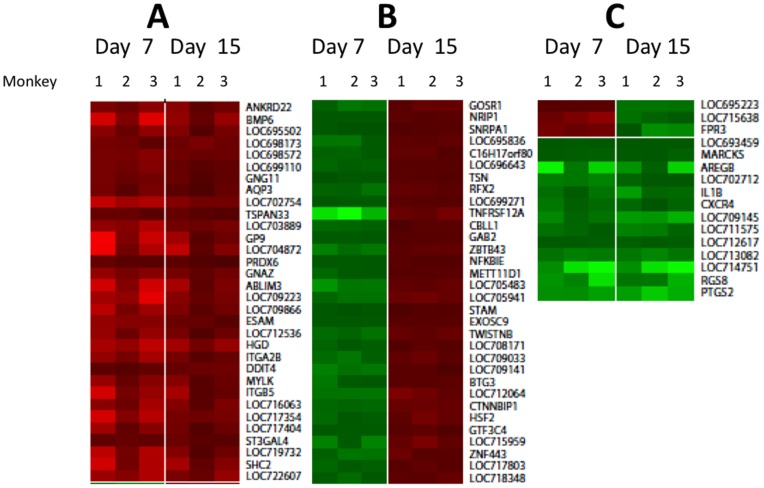
Changes in gene expression in PBMC induced by hep-ATIII treatment of chronically SIV-infected rhesus macaques. The GeneChip® Rhesus Macaque Genome Array was used to assay genome-wide RNA expression (47,000 transcripts) in rhesus macaque PBMC before and after hep-ATIII treatment (n = 3). Differential gene expression comparison of time points when lentiviral replication was observed (day 7) and after viral replication returned to baseline (day 15) for the three monkeys are shown. Only loci or genes that were significantly (P<0.01, ΔΔC_t_ method) up-regulated (red) and down-regulated (green) in comparison to pre-treatment controls are shown. A 3-step color contrast for low, medium and high gene expression change was used. (**A**) Group of genes significantly up-regulated at the day 7 and 15 day time points compared to pre-treatment controls. (**B**) Genes significantly down-regulated at day 7 time point but significantly up-regulated at the day 15 time point compared to pre-treatment controls. (**C**) Genes either significantly up-regulated at the 7 day time point and down-regulated at the 15 day time point, or else significantly down-regulated at both time points compared to pretreatment controls. Full names of genes are given in **Table** **S1**.

The exact molecular mechanisms by which hep-ATIII exerts its anti-HIV effect *in vivo* is unknown. We used transcriptional profiling of PBMC taken from SIV-infected, hep-ATIII-treated rhesus macaques to identify host molecular pathways that might contribute to viral suppression in an extension of our prior *in vitro* investigations [9]. We compared the transcriptional profiles of 47,000 mRNAs at day 7 post-hep-ATIII therapy to the pre-treatment controls, when SIV inhibition was maximal. We also compared transcriptional profiles from day 15 post-therapy, when SIV inhibition had dissipated, to pre-treatment controls. We found that the expression of only a limited number of genes were significantly affected (*P*<0.01, ΔΔC_t_ method) by hep-ATIII therapy when compared to pretreatment (**Fig. 6**). We grouped genes into three groups dependent on the gene-expression pattern at the treatment time point compared to pre-treatment **(** **Fig. 6A** **/B/C**; abbreviations of gene names are specified in **Table S1)**. (1) We identified 18 genes and 15 gene loci that were over-expressed at both day 7 and day 15 in relation to pretreatment **(** **Fig. 6A** **)**. (2) We found 20 genes and 12 loci that were significantly down-regulated at the time of maximal inhibition (day 7) compared to pre-treatment controls, but were up-regulated by day 15 compared to pre-treatment controls, when inhibition had diminished **(** **Fig. 6B** **)**. (3) One gene and 2 loci were up-regulated after 7 days but down-regulated at day 15 **(** **Fig. 6C** **)**. We also found that six genes and 7 loci were down regulated at both time points after therapy (**Fig. 6C**).

### Network Analysis of Hep-ATIII-induced Interactomes during Lentiviral Replication *in vivo*

Interactomes describe relationships between genes that may be functionally linked. Certain genes may be central to the interactome, and modulation of these key genes may exert disproportionate effects on the other genes in the interactome. In order to identify genes that were central to the activity of hep-ATIII, we have previously analyzed the expression of 84 key genes of certain pathways in hep-ATIII treated, HIV-infected hPBMC *in vitro*, and found NFκB and prostaglandin synthetase-2 (PTGS2) to be highly regulated by hep-ATIII [9]. In the present study, we extended our analysis to a whole genome expression profile of PBMC from hep-ATIII-treated, SIV-infected rhesus macaques, evaluating 47,000 transcripts in all.

**Figure 7:**
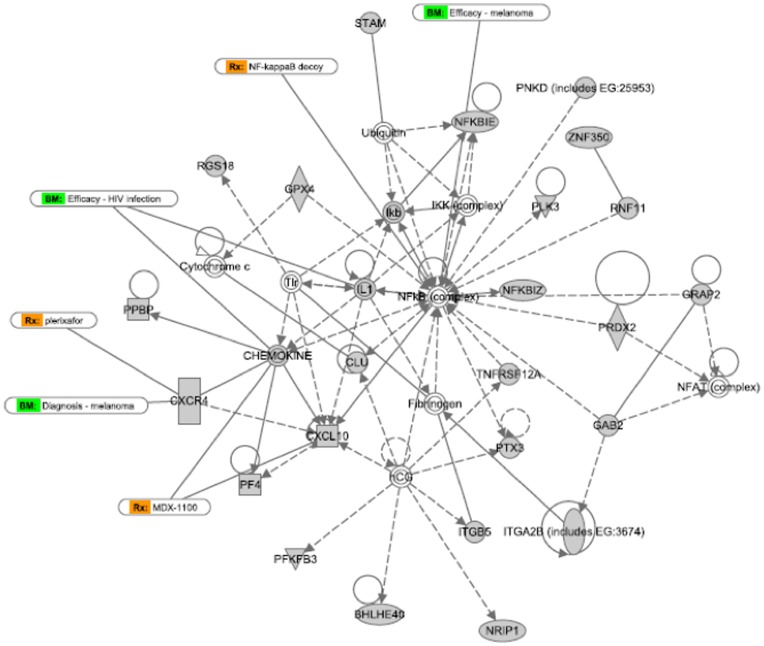
Highest scoring network after interactive network analysis of gene expression changes induced after hep-ATIII treatment of SIV-infected rhesus macaques. The highest scoring primary transcriptional network activated by hep-ATIII treatment of chronically infected rhesus macaques, at a time point when viral replication is inhibited by hep-ATIII (day 7), is shown. Rx (orange): potential medication treatment options, BM (green): possible biomarkers. Network analysis was performed using Ingenuity 8.0 software. Explanation for symbols is given in **Figure S2**.

**Figure 8:**
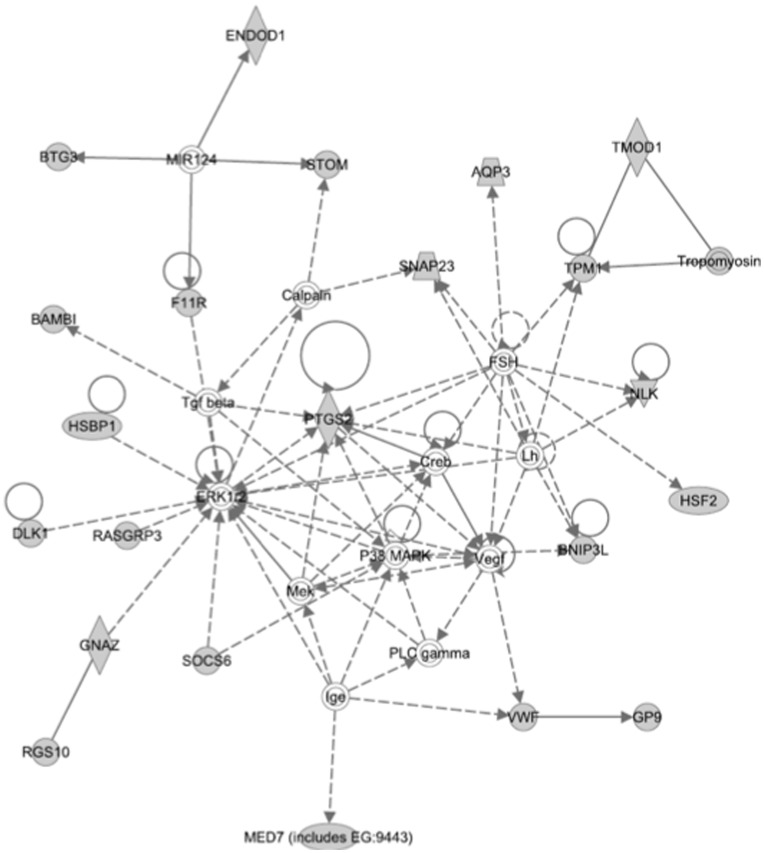
Second-highest scoring network after interactive network analysis of gene expression changes induced after hep-ATIII treatment of SIV-infected rhesus macaques. The second-highest scored network activated by hep-ATIII treatment of chronically SIV-infected rhesus macaques, at a time point when viral replication is inhibited by hep-ATIII (day 7), is shown. Network analysis was performed using Ingenuity 8.0 software. Explanation for symbols is given in **Figure S2**.

Applying Ingenuity-based network analysis to these gene expression profiles we once again identified NFκB in one network and ERK1/2 and PTGS2 in another network central to the two highest scoring regulatory networks modulated by hep-ATIII treatment (**Fig. 7** **,** **Fig. 8** **, Fig. S1** contains explanation of network symbols). Thus, in this comprehensive analysis of transcriptional activation by hep-ATIII, we have confirmed that NFκB and ERK1/2-PTGS2 are the likely major effectors of hep-ATIII activity as described for an *in vitro* system with hPBMC before [9].

## Discussion

Serpins are induced rapidly following virus infection as part of a complex host innate immune response [7]. Mounting clinical evidence demonstrates an association between increased levels of serpin expression and either reduced HIV acquisition in uninfected individuals or delayed disease progression in chronically infected individuals [12], [13], [14], [51], [52], [53], [54], [55]. For example, serpins have been found to be present at high levels in the cervical fluids of uninfected but repeatedly HIV-1 exposed sex workers [56]. ATIII, a serpin with functions in the coagulation cascade, was shown to have antiviral activity *in vitro* against not only HIV but HCV and HSV as well [9], [14], [15], [16], [57]. We are beginning to recognize that the serpins may have broad roles in the innate immune response, which in the case of ATIII includes an anti-inflammatory function in sepsis [58], anti-angiogenesis in tumor growth [59], and chemotaxis for neutrophils, human peripheral blood lymphocytes and monocytes [60], [61], [62]. The role of serpins as adjuvants of the innate immune system may suggest a potentially novel application for serpins in antiviral therapy.

Although the arsenal of small molecule HIV inhibitors continues to grow, drug resistance remains an important obstacle to long-term HIV therapy. Modulators of the innate immune system are attractive therapeutics because they act indirectly on the virus through multiple host pathways, and so are not as vulnerable to the evolution of viral resistance mutations. Indeed, our results suggest that ATIII may be an effective part of a salvage regimen for patients with highly drug resistant HIV strains. We also found that when appropriately modified and targeted through liposomal encapsulation, hep-ATIII appears to be very safe, with a favorable TI >100 and no obvious negative effects in murine and nonhuman primate models.

Our experiments suggest that the precise biochemical modification and packaging of ATIII is critical to its therapeutic utility. It is well described that the various biological functions of ATIII are dependent on its tertiary structure [59], [63]. This structure-dependent functionality of ATIII holds true for its ability to inhibit HIV as well. Interestingly, *in vitro*, heparin-activated ATIII and the thrombin-ATIII complex showed the highest level of HIV-1 inhibition, followed by pre-latent ATIII [15], [16]. A relaxed form of ATIII has a 50% reduced inhibitory activity, whereas HIV inhibition *in vitro* is negligible for the L-isoforms of ATIII [14]. *In vivo* as well, ATIII antiviral activity appears to be dependent on biochemical modification: CD8^+^ T cells of HIV long-term non-progressors (LTNP) exhibit enhanced ability to activate ATIII that may be partially responsible for the reported non-cytolytic inhibition of HIV-1 in this cohort [64].

ATIII is predominately in its S-configuration in blood at a physiological concentration of about 2.4 µM. To determine whether modification of ATIII has an effect on *in vivo* therapeutic HIV activity, we assessed three forms of the protein: non-activated ATIII, heparin-activated ATIII - both given intravenously - and liposomally encapsulated ATIII given subcutaneously. Non-activated ATIII, at a concentration sufficient to reduce inflammation in a baboon model of sepsis [34], and at 10–20-fold normal physiologic concentrations, did not impede viral replication. *In vitro* experiments had demonstrated that the anti-HIV activity of ATIII could be enhanced through heparin activation [16], and in concordance with this we found that intravenous administration of hep-ATIII resulted in a modest inhibition of viral replication *in vivo*, confirming the importance of ATIII activation.

However, we observed the most potent inhibition of plasma virus when ATIII was packaged in immunoliposomes and delivered subcutaneously. There are several possible explanations for this observation: (1) It is likely that unencapsulated hep-ATIII is not specifically transported to lymph nodes, a tissue that harbors viral replication [65], while in contrast, anti-HLA-DR immunoliposomes likely transport ET-ATIII to this location [19]. (2) Follicular dendritic cells (FDC) may stimulate viral replication in lymphocytes, and it has been demonstrated that serpins may interfere with this process [66]. (3) HIV disease progression is associated with ATIII deficiency in blood [51], [52], [67]. Nevertheless, there is likely considerable circulating anti-HIV serpin activity in plasma, such that the serpin anti-HIV activity in plasma may be saturated, and intravenous inoculation of hep-ATIII into the plasma has limited additive effect. In contrast subcutaneous administration of ET-ATIII targeting lymphoid organs maybe more effective because baseline serpin activity in lymphoid organs is minimal.

We probed the underlying mechanism of hep-ATIII anti-HIV activity using software-supported interactome analysis, which allowed us to identify key host cell factors that are immediately downstream of drug treatment, and which in turn modulate the expression of overarching gene networks. We previously found that hep-ATIII activated two interactomes in HIV-1 infected PBMC: one interactome dependent on the NFκB transcription factor and a second interactome anchored by ERK1/2. These host factors are known to significantly impact HIV-1 replication. We now have expanded our analysis of transcriptional changes downstream of treatment with hep-ATIII, and studied these changes in the significantly more complex *in vivo* environment. Once again we found networks regulated by NFκB **(** **Fig. 7** **)** and ERK1/2-PTGS2 **(** **Fig. 8** **)** associated with hep-ATIII treatment confirming earlier *in vitro* results from PBMC acutely infected with HIV-1 [9]. There is a great need to counter HIV-induced inflammation and its consequences on the central nervous system (CNS) including HIV dementia. In the current studies we found that hep-ATIII treatment down-regulated NFκB after 7 days. This is important since the NFκB dimer consisting of p50 and RelA is considered to be the largest contributor to activation of HIV transcription and inflammation [68].

Our second network was centered around ERK1/2 and seems to be dependent on PTGS2, an HIV inhibitory host cell factor described earlier [9]. Thus, another possible mechanism by which hep-ATIII might prevent HIV-induced dementia is through its anti-inflammatory effect since prostaglandins were found to block inflammation through inhibition of HIV-1 Tat-mediated ERK1/2 activation [69].

There are several limitations to these studies. (1) In our investigation of virus-induced cytotoxicity in the spleens of humanized mice, we have not characterized the specific cellular populations that are preserved in contrast to those that are lost, nor have we determined the mechanism by which hep-ATIII may prevent cytotoxicity. (2) We have not provided a mechanism for viral rebound after treatment with ET-ATIII. Our gene expression analyses suggest that the mechanism of ET-ATIII is through recruitment of innate antiviral mechanisms. Although it is unclear whether HIV can rapidly evolve resistance to host innate factors in such a rapid timeframe, we suspect that the rebound is most likely due to the clearance of ET-ATIII from the host, and hence loss of its suppressive activity. (3) Our non-human primate pilot studies are limited by the number of animals available, but we believe provide justification for a larger scale trial. Clearly testing a more prolonged administration regimen is needed to more fully evaluate the safety and efficacy of ET-ATIII as an anti-HIV therapeutic.

In conclusion, our data suggest that activated ATIII targeted to lymph nodes may have substantial *in vivo* activity against HIV-1. Further understanding of the mechanisms by which hep-ATIII interferes with HIV replication in lymphoid tissues might have important implications for the design of therapeutic strategies that harness the innate immune system for both its direct antiretroviral potential and its ability to modulate the adaptive immune response.

## Supporting Information

Figure S1 **Legend to interactive networks.** Explanation of symbols and lines from Figure 7 and Figure 8.
(TIF)

Table S1 **Full names of genes significantly altered by hep-ATIII treatment used in** **Figure 6** **.**
(DOCX)
