# Supplementary figures and images for: In Vivo Anti-HIV Activity of the Heparin-Activated Serine Protease Inhibitor Antithrombin III Encapsulated in Lymph-Targeting Immunoliposomes
Source: PLoS One. 2012 Nov 2;7(11):e48234. doi: 10.1371/journal.pone.0048234 (PMC3487854; doi:10.1371/journal.pone.0048234)

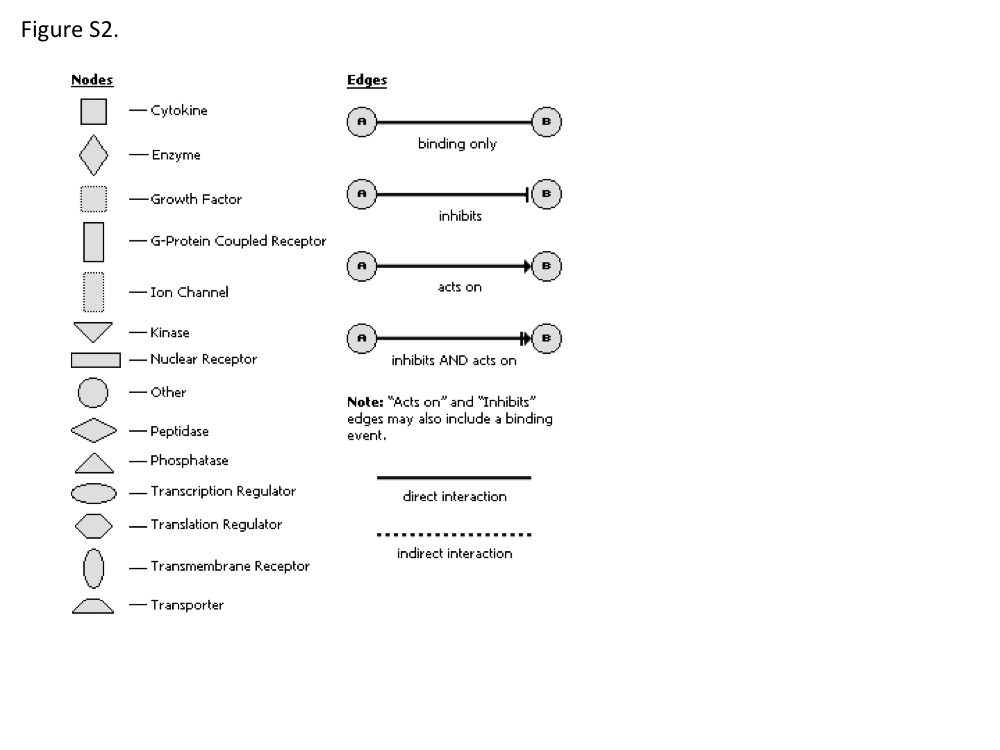

Supplement: Figure S1 — Legend to interactive networks. Explanation of symbols and lines from Figure 7 and Figure 8. (TIF) [file pone.0048234.s001.tif]
