# Supplementary material for: In Vivo Anti-HIV Activity of the Heparin-Activated Serine Protease Inhibitor Antithrombin III Encapsulated in Lymph-Targeting Immunoliposomes
Source: PLoS One. 2012 Nov 2;7(11):e48234. doi: 10.1371/journal.pone.0048234 (PMC3487854; doi:10.1371/journal.pone.0048234)
Supplement: Table S1 — Full names of genes significantly altered by hep-ATIII treatment used in Figure 6 . (DOCX) [file pone.0048234.s002.docx]

**Table S1. Full names of genes significantly altered by hep-ATIII treatment used in Figure 6**

| **Gene ID** | **Gene name** |
| --- | --- |
| ANKRD22 | Ankyrin repeat domain 22 |
| BMP6 | Bone morphogenetic protein 6 |
| GNG11 | Guanine nucleotide-binding protein G(I)/G(S)/G(O) subunit gamma-11 |
| AQP3 | Aquaporin 3 |
| TSPAN33 | Tetraspanin 33 |
| GP9 | Glycoprotein IX |
| PRDX6 | Peroxiredoxin-6 |
| GANZ | Guanine nucleotide binding protein (G protein), alpha z polypeptide |
| ABLIM3 | Actin binding LIM protein family, member 3 |
| ESAM | Endothelial cell-selective adhesion molecule |
| HGD | Homogentisate 1,2-dioxygenase |
| ITGA2B | Integrin, alpha 2b (platelet glycoprotein IIb of IIb/IIIa complex, antigen CD41) |
| DDIT4 | DNA-damage-inducible transcript 4 protein |
| MYLK | Myosin light chain kinase 4 |
| ST3GAL4 | ST3 beta-galactoside alpha-2,3-sialyltransferase 4 |
| SHC2 | SHC (Src homology 2 domain containing) transforming protein 2 |
| GOSR1 | Holgi SNAP receptor complex member 1 |
| NRIP1 | Nuclear receptor-interacting protein 1 |
| SNRPA1 | Small nuclear ribonucleoprotein polypeptide A' |
| C16H17orf80 | Uncharacterized protein C17orf80-like |
| TSN | Translin |
| CBLL1 | c-Cbl-like protein 1 |
| GAB2 | Growth factor receptor-bound protein-associated binder 2 |
| ZBTB43 | Zinc finger and BTB domain containing protein 43 |
| NFKBIE | Nuclear factor of kappa light polypeptide gene enhancer in B-cells inhibitor, alpha |
| METT11D1 | Methyltransferase 11 domain containing 1 |
| STAM | Signal-transducing adaptor molecule |
| EXOSC9 | Exosome component 9 |
| TWISTNB | Twist neighbor protein |
| BTG3 | B-cell translocation gene |
| CTNNBIP1 | Catenin, beta interacting protein 1 |
| HSF2 | Heat shock factor protein 2 |
| GTF3C4 | General transcription factor 3C polypeptide 4 |
| ZNF443 | Zinc finger protein 443 |
| FPR3 | Formyl peptide receptor 3 |
| MARCKS | Myristoylated alanine-rich protein kinase C substrate |
| AREGB | Amphiregulin B |
| IL1B | Interleukin-1 beta |
| CXCR4 | C-X-C chemokine receptor type 4 |
| RGS8 | Regulator of G-protein signaling 8 |
| PTGS2 | Prostaglandin-endoperoxide synthase 2 |
